# High-Risk Neuroblastoma with Metastases to Bilateral Kidneys at Diagnosis

**DOI:** 10.1155/2017/5375091

**Published:** 2017-03-30

**Authors:** Toshihide Yoshikawa, Akihiko Tanizawa, Koji Suzuki, Kazumi Ikeda, Eishi Nomura, Yumekichi Maeda, Nanae Tanaka, Kenta Yamada, Yasuhiro Sakai, Yoshiaki Imamura, Yusei Ohshima

**Affiliations:** ^1^Department of Pediatrics, Faculty of Medical Sciences, University of Fukui, 23-3 Matsuokashimoaizuki, Eiheiji-cho, Yoshida-gun, Fukui 910-1193, Japan; ^2^Department of Human Resource Development for Cancer, Faculty of Medical Sciences, University of Fukui, 23-3 Matsu-okashimoaizuki, Eiheiji-cho, Yoshida-gun, Fukui 910-1193, Japan; ^3^Department of Pediatrics, Japanese Red Cross Fukui Hospital, 2-4-1 Tsukimi, Fukui 918-8011, Japan; ^4^Department of Tumor Pathology, Faculty of Medical Sciences, University of Fukui, 23-3 Matsuokashimoaizuki, Eiheiji-cho, Yoshida-gun, Fukui 910-1193, Japan; ^5^Division of Surgical Pathology, University of Fukui Hospital, 23-3 Matsuokashimoaizuki, Eiheiji-cho, Yoshida-gun, Fukui 910-1193, Japan

## Abstract

Renal metastasis at diagnosis with neuroblastoma is rare. We present a 14-month-old boy who was diagnosed with high-risk neuroblastoma with multiple metastases, including bilateral kidneys. He received five cycles of induction chemotherapy and high-dose chemotherapy with autologous peripheral blood stem cell transplantation. All of the lesions shrank, and magnetic resonance imaging indicated that some of the metastases had disappeared. However, there were residual masses in the bilateral kidneys, and histological examination revealed the presence of tumor cells. Therefore, the patient underwent unrelated cord blood stem cell transplantation, which involved killer-ligand incompatibility in the graft-versus-host direction, in addition to human leukocyte antigen C and DRB1 mismatches. Three months later, tumor progression occurred from the residual mass in the sacral canal and a new lesion in the pancreas. Although tumor progression could not be controlled by additional chemotherapy and local radiotherapy, the metastatic nodules in bilateral kidneys did not increase in size before his death. To the best of our knowledge, this is the first report of neuroblastoma with bilateral renal metastases in the English medical literature. In addition, this case suggests that the combination of chemotherapy and immunotherapy may inhibit the progression of the renal lesions under certain conditions.

## 1. Introduction

Renal metastasis at diagnosis with neuroblastoma is extremely rare. The published incidences ranged from 0% (none of 567 cases) [1] to 0.7% (1 of 153 cases) [2] in stage 4 patients (excluding stage 4S cases), whereas the incidences at other sites were 75.7% in bone marrow, 63.7% in bone, 34.0% in lymph node, 22.4% in liver, and 20.8% in intracranial and orbital sites [1]. Since few case reports of neuroblastoma with renal metastasis have been published [3, 4] and, to the best of our knowledge, there are no reports in the English medical literature of neuroblastoma with bilateral renal metastases at diagnosis, the significance of the condition for prognosis remains unknown. In the setting of bilateral and multiple renal metastases, local therapeutic modalities for the kidneys, such as nephrectomy and/or radiotherapy, cannot be considered because the patient will completely lose renal function. Here, we present the clinical course of a patient with stage 4 neuroblastoma who had multiple metastatic lesions in bilateral kidneys at diagnosis.

## 2. Case Presentation

**Figure 1:**
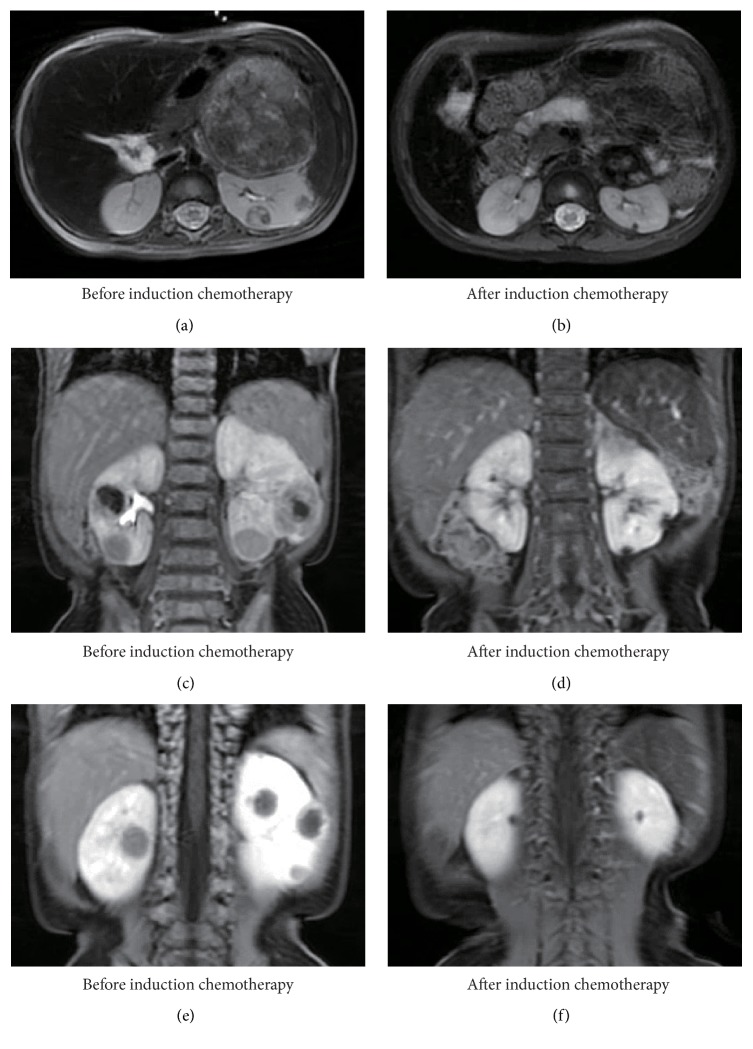
MRI findings before and after induction chemotherapy. Before induction chemotherapy, the T2-weighted axial image (a) and contrast enhanced coronal images (c, e) revealed a left adrenal mass and multiple bilateral renal masses. After induction chemotherapy, the T2-weighted axial image (b) and contrast enhanced coronal images (d, f) showed that the primary tumor and all lesions in bilateral kidneys had decreased in size.

A 14-month-old boy with a large abdominal mass was admitted to our hospital. Serum neuron-specific enolase (NSE) was markedly elevated (1,000 ng/mL), while urinary homovanillic and vanillylmandelic acid were within the normal range. Magnetic resonance imaging (MRI) and computerized tomography revealed a left adrenal tumor with calcification and metastases to cranial bones, cranial base, bilateral orbits, left pleura, thoracic paravertebral soft tissue, para-aortic lymph nodes, soft tissue in the sacral canal, and bilateral kidneys (Figures 1(a), 1(c), and 1(e)). However, blood and urine tests indicated no impairment of renal function. Uptake of iodine-123-metaiodobenzylguanidine (^123^I-MIBG) was heterogeneous, being highly increased in cranial bones, cranial base, and bilateral orbits but only slightly increased in the primary lesion and bilateral kidneys and weak in other sites. Biopsy of the adrenal tumor was performed, and a diagnosis of poorly differentiated neuroblastoma with low mitosis-karyorrhexis index was confirmed. NSE immunohistochemistry was partially positive. Overall, histology was favorable based on International Neuroblastoma Pathology Classification; however, fluorescence in situ hybridization showed that tumor cells with and without amplification of the* MYCN* oncogene coexisted in the biopsied specimen. Bone marrow examination showed tumor cells with a highly amplified* MYCN* oncogene. The patient was diagnosed as high-risk according to the International Neuroblastoma Risk Group Classification System.

**Figure 2:**
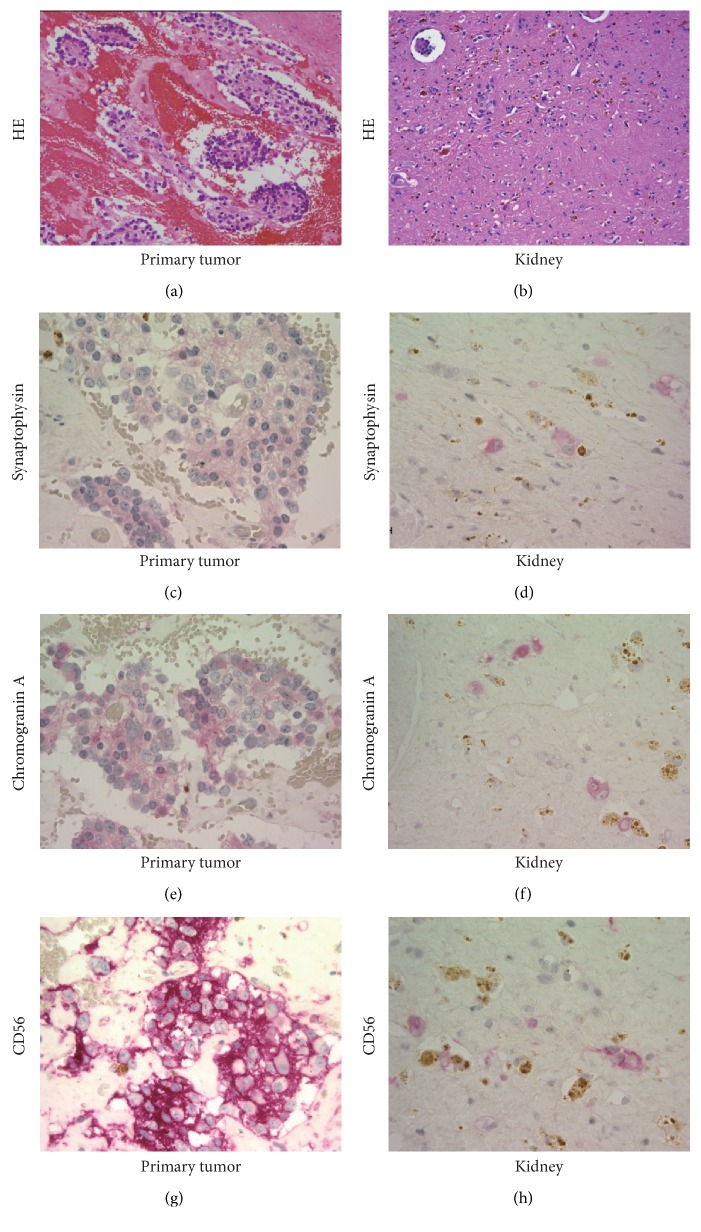
Histological findings of the primary tumor and a residual mass in the left kidney. In the primary tumor, H&E staining demonstrated neuroblastoma cell clusters (a), and these cells were immunohistochemically positive for (c) synaptophysin, (e) chromogranin A, and (g) CD56. In the left kidney, it was difficult to identify the metastatic lesion due to chemotherapy (b); however, immunohistochemistry revealed the individually scattered neuroblastoma cells (d–h). Note that color reaction was performed using new fuchsin (red), and brown pigments were hemosiderin or ceroid.

The patient received five cycles of induction chemotherapy, high-dose chemotherapy (HDC) with autologous peripheral blood stem cell transplantation (auto-PBSCT), followed by surgical resection of the primary tumor together with renal biopsy, and finally cranial irradiation (Table 1). Although all of the lesions shrank and serum NSE decreased to within the normal range, there were still residual masses in the primary site, left orbit, sacral canal, and bilateral kidneys after five courses of induction chemotherapy (Figures 1(b), 1(d), and 1(f)). The sizes of residual masses in the primary lesion, sacral canal, and bilateral kidneys were slightly reduced by HDC. Histological examination of the resected primary tumor and a biopsy specimen obtained from the left kidney were positive for synaptophysin, chromogranin A, and cluster of differentiation (CD) 56 but not NSE, confirming the presence of tumor cells (Figures 2(a)–2(h)). These findings supposed that some tumor cells also remained in the opposite renal metastatic nodules, which were depicted in the same manner on MRI. We chose the graft-versus-tumor (GVT) approach with allogeneic hematopoietic stem cell transplantation (allo-HSCT) as a potentially curative treatment to avoid bilateral nephrectomy or local radiotherapy to both kidneys. The patient underwent unrelated cord blood stem cell transplantation (CBT) from a human leukocyte antigen- (HLA-) C and DRB1 mismatched donor at four months after auto-PBSCT (Table 1). In addition, the HLA-C mismatch between the donor and the recipient induced killer cell immunoglobulin-like receptor- (KIR-) ligand incompatibility in the graft-versus-host direction to generate natural killer (NK) cell alloreactivity against neuroblastoma cells. The conditioning regimen consisted of fludarabine, melphalan, and total body irradiation (TBI), and tacrolimus with short-term methotrexate was administrated for graft-versus-host disease (GVHD) prophylaxis (Table 1). Radiotherapy to the primary tumor bed and sacrum was planned. However, the residual mass in the sacral canal was enlarged and a new metastatic lesion appeared in the pancreatic tail at three months after CBT. Tumor progression could not be controlled by additional chemotherapy and local radiotherapy, and the patient succumbed at 26 months after diagnosis and 12 months after relapse. There was no recurrence in cranial bones where 19.8 Gy of local radiotherapy was delivered between HDC and CBT. Furthermore, the metastatic nodules in bilateral kidneys did not increase in size before his death and there was no evidence of GVHD. We could not examine the histopathological status of the renal involvements because autopsy was not performed.

## 3. Discussion

Multidisciplinary treatment has improved therapeutic efficiency for high-risk neuroblastoma [5, 6]. However, the prognosis for patients with renal metastasis at diagnosis is unclear, since kidney is a rare metastatic site [1, 2]. When the sites of renal metastases were unilateral, surgical resection or radiotherapy for the involved kidney is an attractive option, whereas both modalities could not be considered in our case with bilateral and multiple renal metastatic lesions.

In the 2000s, allo-HSCT was reported to induce a GVT effect against neuroblastoma [7–9]. Moreover, lacking an HLA ligand for inhibitory KIR was reported to be associated with low risk of disease progression, even in patients who had undergone autologous HSCT for high-risk neuroblastoma [10]. Therefore, allogeneic immunity and NK cell alloreactivity in the graft-versus-host direction to tumor cells were also expected in our case (Table 1). In this case, although new and recurrent lesions were detected in multiple sites within three months of CBT, the renal lesions did not progress. CD56 and perforin expression distinguishes two subsets of human NK cells: CD56^dim^perforin^high^ (cytotoxic) and CD56^bright^perforin^low^ (noncytotoxic). Carrega et al. reported that the frequencies of total NK cells among lymphocytes and CD56^dim^perforin^high^ NK cells among total NK cells were higher in kidney than in lymph node and stomach [11]. Thus, cytotoxic NK cells may be particularly abundant in the kidney. Furthermore, biological characteristics, including sensitivity to chemotherapy, might be lesion dependent because NSE immunohistochemistry in the primary lesion, uptake of ^123^I-MIBG in the primary lesion and metastatic sites, and* MYCN* amplification in the primary lesion and bone marrow aspirate were heterogeneous, and there was a higher residual tumor burden in the primary lesion compared with the renal lesion after auto-PBSCT (Figures 2(a)–2(h)). Thus, the distribution of NK cells and the biological characteristics of neuroblastoma cells in the renal lesion might contribute to a favorable therapeutic response.

In summary, to the best of our knowledge, this is the first case report of neuroblastoma with bilateral renal metastases at diagnosis in the English medical literature. No clinical progression of the renal lesions occurred during multidisciplinary treatment. Although the patient eventually died, our report suggests that the combination of chemotherapy and immunotherapy may prevent the progression of renal lesions under certain conditions, since 2 Gy of TBI at CBT is not usually enough to control neuroblastoma progression. This case shows the possibility that chemotherapy and immunotherapy might be therapeutic options against neuroblastoma with bilateral renal involvement to preserve renal function, if the primary tumor and other metastatic involvements could be controllable with multidisciplinary treatment including surgical interventions.

## Figures and Tables

**Table 1:** Details of chemotherapy and CBT.

Induction chemotherapy				
Cyclophosphamide	1,200 mg/m^2^/day on days 1 and 2, except for day 2 in cycle 1			
Vincristine	1.5 mg/m^2^ on day 1			
Pirarubicin	40 mg/m^2^ on day 3			
Cisplatin	20 mg/m^2^/day continuously from days 1 to 5			
HDC with auto-PBSCT				
Melphalan	100 mg/m^2^/day on days -9 and -8			
Etoposide	200 mg/m^2^/day on days -7 to -4			
Carboplatin	400 mg/m^2^/day continuously on days -7 to -4			
Cord blood transplantation				
Conditioning regimen				
Fludarabine	25 mg/m^2^/day on days -7 to -3			
Melphalan	70 mg/m^2^/day on days -4 and -3			
TBI	2 Gy on day -2			
GVHD prophylaxis	Tacrolimus and short-term methotrexate			

HLA typing	A	B	C	DRB1

Recipient	24:02/—	51:01/52:01	12:02/14:02	04:05/15:02
Donor	24:02/—	51:01/52:01	12:02/15:02	15:02/—

CBT: cord blood stem cell transplantation; HDC: high-dose chemotherapy; auto-PBSCT: autologous peripheral blood stem cell transplantation; TBI: total body irradiation; GVHD: graft-versus-host disease; HLA: human leukocyte antigen.
